# Analysis of injured-skin SS-OCT images based on combined attention UNet

**DOI:** 10.1371/journal.pone.0324327

**Published:** 2025-07-11

**Authors:** Xiyu Zheng, Jingyuan Wu, Qiong Ma, Diantao Luo, Qingyu Cai, Haiyang Sun, Hongxing Kang

**Affiliations:** 1 College of Information Engineering, Henan University of Science and Technology, Luoyang, Henan, China; 2 Beijing Institute of Radiation Medicine, Beijing, China; 3 College of Life Sciences, Hebei University, Baoding, Hebei, China; Islamia University of Bahawalpur: The Islamia University of Bahawalpur Pakistan, PAKISTAN

## Abstract

Optical coherence tomography (OCT) is a noninvasive imaging technique that provides high-resolution images of superficial skin tissues and has become widely used for diagnosing various skin disorders. Assessing laser-induced skin tissue damage is essential for understanding the healing mechanisms and optimizing treatment strategies. However, effectively quantifying skin damage and its correlation with laser dosage and recovery time poses a challenge. In this study, we established a laser-induced skin injury model in mice, utilizing 1 μm–2 μm laser wavelengths. We obtained SS-OCT images of the injury site under different laser doses and recovery times. To enhance image clarity, we applied noise reduction using the BM3D algorithm. We employed an improved UNet network model that incorporates SimAM and PSA modules, forming three attention mechanisms: TandemAT-UNet, ParallelAT-UNet, and NestedAT-UNet. These models were used to segment the damaged skin regions, followed by a 3D reconstruction method to quantitatively evaluate the volume of skin damage while analyzing changes about laser dose and recovery time.The BM3D algorithm effectively suppressed high-noise components, significantly improving image clarity. Among the three models, ParallelAT-UNet exhibited the best segmentation performance, achieving a Dice coefficient of 0.9364, mean Pixel Accuracy (mPA) of 92.67%, mean Intersection over Union (mIoU) of 96.31%, and an accuracy of 99.39%. Quantitative analysis revealed that laser doses between $25.0\;{\text{J/cm}}^{2}$ and $36.5\;{\text{J/cm}}^{2}$ caused minimal changes in skin damage volume, while doses ranging from $44.2\;{\text{J/cm}}^{2}$ to $74.4\;{\text{J/cm}}^{2}$ resulted in significant changes, which varied according to both the dose and recovery time. All groups showed signs of healing by 14 days post-laser treatment, with damage volumes smaller than the initial values. This study presents an efficient and reliable method for the quantitative assessment of laser-induced skin damage using OCT imaging. The findings demonstrate a strong relationship between laser dosage, recovery time, and skin damage, highlighting potential applications for noninvasive diagnosis and treatment monitoring using OCT.

## Introduction

Since its introduction in 1960, lasers have been widely used in various fields such as industry, communication, and medical treatment due to their advantages of high brightness, good coherence, and excellent directionality. Today, laser technology has permeated all aspects of social production and life, becoming a universal technology across multiple domains [1]. However, as the application of laser technology has become increasingly widespread, the issue of laser damage has garnered greater attention [2], leading to the establishment of various laser damage assessment methods [3–6]. As the largest organ in the human body, skin is one of the primary targets of laser thermal damage. The degree of laser-induced thermal damage to the skin is closely related to the laser’s power and irradiation time [7–9]. Therefore, exploring the quantitative relationship with the extent of thermal damage is crucial for establishing laser protection protocols and clinical treatments.

Optical coherence tomography (OCT) is a non-invasive imaging technique based on the principle of low-coherence light, generating high-resolution tomographic images by measuring the echo time and intensity of reflected light [10]. In the medical field, OCT is widely used in ophthalmology and cardiovascular disciplines due to its noninvasive nature, real-time capabilities, and high sensitivity [11,12]. In recent years, an increasing number of studies have demonstrated that OCT is also effectively utilized in dermatology [13]. Several studies have shown that OCT serves as a valuable noninvasive modality for detecting areas of skin thermal damage. Fan *et al*. [14] employed OCT in combination with dermoscopy and pathology to evaluate the localized segmentation of thermal injuries in the dermis, adipose, and muscle layers of skin using a 3.8 μm laser. Xu *et al*. [15] utilized polarization-sensitive optical coherence tomography (PS-OCT) to image skin burn tissue, employing a PCA-based imaging technique to assess the extent of skin burns. This approach included a PCA-based image fusion method that generated enhanced images with improved tissue contrast for differentiating burn extent. Noninvasive real-time imaging with OCT further underscores the advantages of this technology for monitoring and assessing thermal skin injuries compared to traditional methods such as dermoscopy and pathology testing.

The advent of deep learning techniques has greatly enhanced the ability of computers to recognize and process images. Compared to traditional algorithms, deep learning methods can better assist in medical diagnosis [16]. In the medical field, various auxiliary diagnostic and treatment methods based on deep learning have demonstrated practical application value [17–21]. Additionally, OCT images, as a typical form of medical image data, combined with deep learning techniques, offer new approaches for disease diagnosis and treatment [22]. In dermatology, Lin *et al*. [23] proposed a convolutional neural network-based method for measuring skin epidermal thickness, determining the ridge boundaries of the skin surface and the dermal-epidermal junction in fingertip skin cross-sectional optical coherence tomography images. Ji Y *et al*. [24] developed a deep learning-based skin layer segmentation method that automatically and quantitatively assessed epidermal and crusted tissue thickness in vivo in a rodent model, achieving greater accuracy and speed in calculating thickness compared to human experts. Szczepanik *et al*. [25] demonstrated that spectral-domain optical coherence tomography (SD-OCT) could evaluate and differentiate between the rat epidermis and dermis using histological images from healthy male rats. Fischman S *et al*. [26] used deep learning to noninvasively score cellular heterogeneity in keratinocyte carcinoma in 3D LC-OCT images. Breugnot *et al*. [27] developed a model for automatically scoring the quality of the dermal matrix in vivo LC-OCT images relevant to dermatology and aesthetics. Rasel *et al*. [28] compared the differences between 2D and 3D convolutional neural networks for predicting glaucoma in OCT images, integrating deep learning with OCT. Zhu *et al*. [29] proposed a novel interface segmentation technique for corneal layers, successfully segmenting both 2D and 3D OCT images. Mehdizadeh *et al*. [30] developed a new method that combined texture loss with Generative Adversarial Networks (GANs) for denoising optical coherence tomography scans, thereby improving the accuracy of clinical diagnoses. Yang *et al*. [31] employed a weakly-supervised learning approach for retinal OCT images through a novel anomaly bootstrapping mechanism aimed at semantic segmentation. Wang *et al*. [32] applied four common network models to 2 μm laser-induced skin damage images to quantitatively analyze the biological effects of increased damage volume versus radiation dose, confirming the effectiveness of deep learning in laser damage skin image studies.

The development of deep learning techniques has opened up new avenues for medical image detection, and convolutional neural networks, especially UNet, are efficient and accurate in the semantic segmentation of medical images [33–37]. Although OCT techniques have demonstrated significant advantages in skin damage assessment, existing segmentation methods are often difficult to accurately capture the boundaries of complex skin damage regions and are more sensitive to noise, which poses a challenge to the subsequent comprehensive assessment of the relationship between damage changes over time and dose. To this end, we introduce an improved model of UNet that combines SimAM and PSA attentional mechanisms, aiming to achieve accurate segmentation of laser-damaged skin regions and support quantitative analysis by enhancing feature expressivity and noise suppression. The main contributions of this paper are as follows:

(1) Constructed a dataset for laser-injured skin image segmentation. We have acquired and labeled images of laser-induced injury regions in mice at seven different doses and five time points to form a dataset for laser injury skin image segmentation tasks. The dataset covers a rich combination of doses and recovery times, providing high-quality basic data for studying the relationship between skin laser injury and recovery. Experimental results based on this dataset demonstrate the effectiveness and robustness of this paper’s method in damaged skin segmentation.

(2) The combined Attention UNet model for combining multi-attention mechanisms is proposed. We designed the CombinedAT-UNet model by integrating the SimAM and PSA attention modules into the up-sampling stage of UNet. The SimAM module can dynamically calculate the attention weights of pixels to highlight important spatial information, thus enhancing the model’s ability to capture local features; the PSA attention module effectively models global contextual dependencies through a parallel structure to improve the segmentation accuracy of complex background and detail regions. Based on the combination of three different configurations (i.e., TandemAT-UNet, ParallelAT-UNet, and NestedAT-UNet), the comparative experiments verified the significant improvement of the segmentation accuracy of these modules for skin damage. Among them, the best-performing ParallelAT-UNet also achieves excellent results in comparison with the SOTA method.

(3) Quantitative assessment of laser damage changes was realized. We evaluated the volumetric changes of laser-induced injuries at different doses and the healing trend with recovery time by 3D reconstruction and quantitative analysis of the results after network segmentation. The experiments show that the proposed model can not only accurately segment the damage region, but also provide a reliable quantitative index for the study of the laser dose-damage-recovery relationship, which further validates the value of the method in practical applications.

We aim to propose a reliable segmentation method that explores the correlation between the volume of thermal damage caused by laser skin treatments and the irradiation dose and duration. This work will provide essential data and technical support for future studies, as well as promote the application of deep learning based optical coherence tomography (OCT) image analysis in the diagnosis and treatment of dermatological conditions.

## Materials and methods

### Experimental setup and system setup

Five KM mice, weighing 20 g–25 g, were purchased from the Beijing Kexing Laboratory Animal Breeding Center, holding animal production license No. SCXK (Beijing) 2018-0010. The mice were uniformly bred in our laboratory animal center and were routinely kept for 5 days prior to the experiments, during which they were observed to be free of abnormalities. The study received approval from the animal experiment protocol at the Laboratory Animal Center of the Beijing Institute of Radiation Medicine, and all animal experiments were conducted in accordance with IACUC-DWZX-2022-687 guidelines.

After the mice were anesthetized intraperitoneally with 1% pentobarbital sodium, a laser irradiation area with a 2 cm × 4 cm square grid array was drawn on the dorsal skin after depilation, with each square measuring 1 cm on each side, resulting in an overall area of 2 cm × 4 cm. In this experiment, a 1 μm–2 μm laser was employed for skin irradiation damage assessments. The laser power was controlled by a computer program, and the irradiation power was monitored using a PD300-3W-V1 laser power meter. An electronic shutter controlled the laser irradiation time, set at 0.2 s. The laser irradiation doses were divided into eight levels, applied sequentially from the lowest to the highest. The power densities on the right side of the mice, from head to tail, were $25.0\;{\text{J/cm}}^{2}$, $31.1\;{\text{J/cm}}^{2}$, $36.5\;{\text{J/cm}}^{2}$, and $41.2\;{\text{J/cm}}^{2}$, while the doses on the left side, from head to tail, were $51.1\;{\text{J/cm}}^{2}$, $58.9\;{\text{J/cm}}^{2}$, $66.7\;{\text{J/cm}}^{2}$, and $74.4\;{\text{J/cm}}^{2}$, respectively.

**Fig 1:**
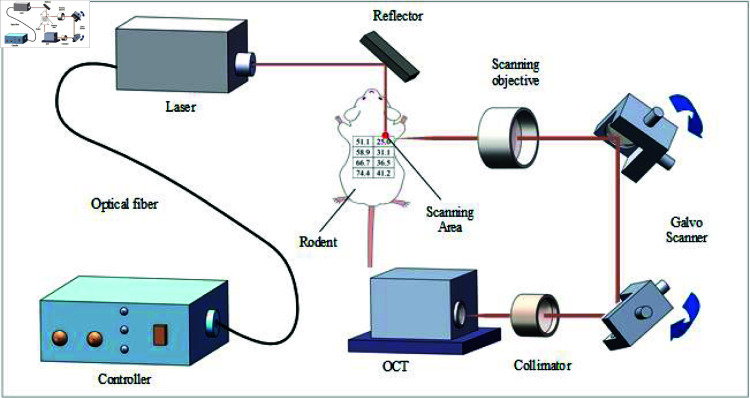
Optical path and SS-OCT imaging system of 1 μm–2 μm laser irradiation on living mouse skin.

At five time points—1 hour, 1 day, 3 days, 7 days, and 14 days post-laser irradiation—the healing of the wound surface was observed and recorded for the five mice, and images were acquired. The image acquisition was performed using a swept-source OCT (SS-OCT) imaging system, which operated at a center wavelength of 1310 nm, a main frequency scan of 100 kHz, and a spectral bandwidth of 91.5 nm. This system achieved a longitudinal resolution of up to 22 μm, a transverse resolution of up to 12 μ, and a temporal resolution of 25 Hz, with an imaging depth of approximately 2.3 mm, generating 200 images per scanning cycle. The optical path of the 1 μm–2 μm laser irradiation on living mouse skin is depicted in Fig 1.

### Deep learning methods

**Fig 2:**
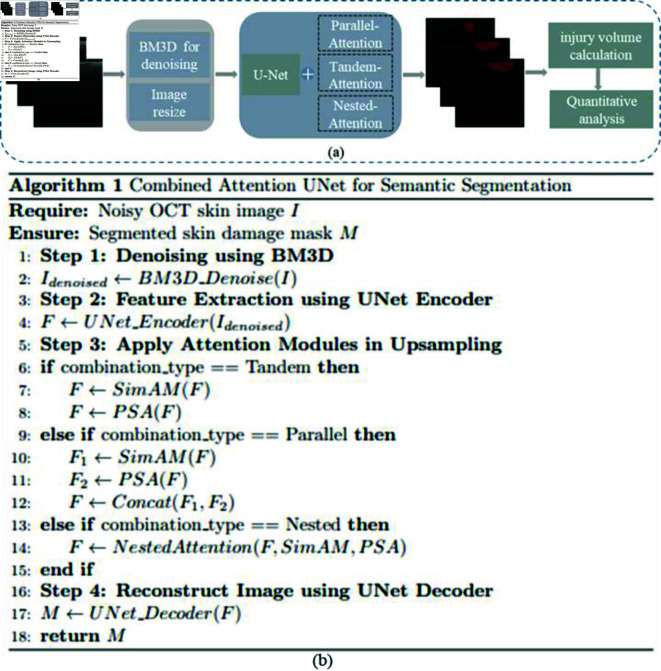
(a) B-Scan skin image damage analysis procedure; (b) B-Scan skin image segmentation algorithm.

UNet is widely utilized in medical image segmentation due to its powerful feature extraction capabilities and effective semantic segmentation. To better address the detailed features of damaged skin and the fusion of features at different scales, a combined attention module was introduced in the upsampling phase of the UNet network during training. In this paper, the B-Scan skin image segmentation program based on deep learning approach in the form of visual flowchart and pseudo-code in Fig 2. , respectively, which employs UNet combined with various attention modules to calculate and evaluate damage after preprocessing the damaged skin images in mice.

#### Dataset preprocessing.

**Fig 3:**
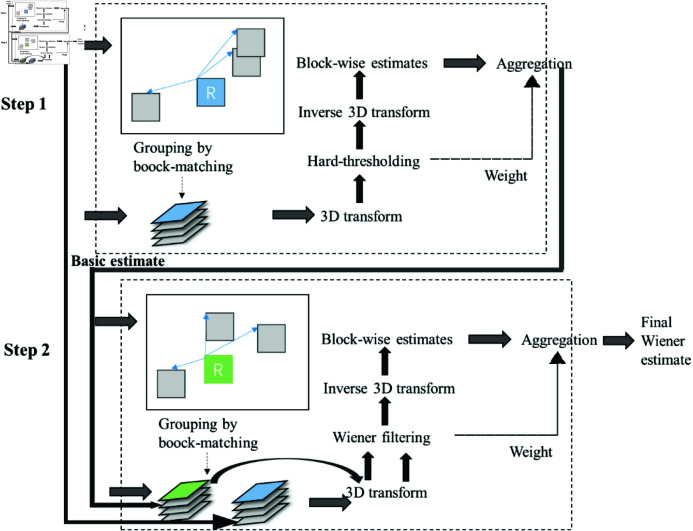
BM3D algorithmic process.

From the OCT image data of mouse skin subjected to different doses of laser damage, 2176 images were selected for preprocessing, which included image denoising and resizing. Scattering noise is a type of multi-coherent noise that is inevitable when imaging with OCT devices; it reduces the contrast of the OCT images and obscures detailed structures, thereby affecting subsequent image processing and analysis. To address this issue, this study employed the BM3D algorithm for noise reduction on the image datasets. BM3D denoising uses a block size of 8 × 8, a search window of 32 × 32, a hard threshold coefficient of 0.1, and a number of iterations of 2. The processing flow is shown in Fig 3. It achieves efficient denoising through block matching and 3D transformation.

The main steps include: first, dividing the image into multiple small blocks and performing similar block matching; second, transforming the similar blocks in 3D and applying hard thresholding to suppress noise; third, inverting the thresholded coefficients and merging the reconstructed blocks back into the image using a weighted averaging method; and finally, repeating the above steps to further enhance the denoising effect. The images were resized to 460 × 460 pixels using OpenCV’s resize method, followed by adjusting the resolution to 512 × 512 pixels using zero-padding. The processed images were then compiled into a network input image dataset, which was divided into training, validation, and test sets in a ratio of 8:1:1.

#### Network structure.

**Fig 4:**
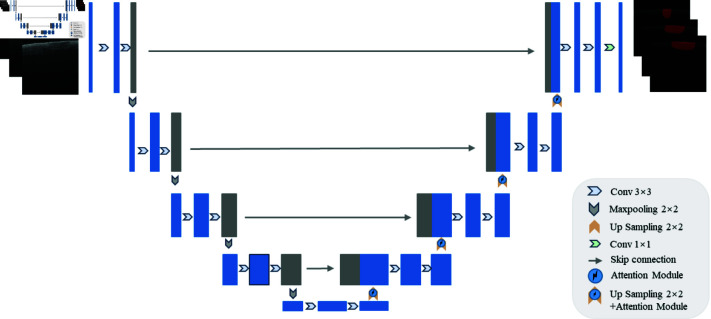
Improved UNet network model with combined attention module.

The attention mechanism enhances the model’s recognition and decision-making abilities in complex tasks by prioritizing key information. By incorporating the attention mechanism into the network, the performance and generalization capabilities of the model can be improved. In this study, to enhance the performance of the UNet model for segmenting skin OCT scan images of laser-damaged regions, different combinations of attention modules were introduced, as illustrated in Fig 4. The attention module primarily utilizes SimAM [38] (Simple Attention Module) and PSA [39] (Pyramid Squeeze Attention) modules. The SimAM module determines the importance of each neuron by optimizing an energy function, enabling the generation of true 3D weights for each neuron directly. This comprehensive attention mechanism captures important feature information more accurately and efficiently; it does not introduce any additional parameters and can be seamlessly integrated into any existing convolutional neural network without adding significant computational complexity or cost. The PSA module processes features at different scales, finely tuning the feature representations to adapt to complex spatial changes. It effectively captures and represents the spatial relationships between features through the pyramid squeezing attention mechanism, thereby improving segmentation accuracy. Additionally, the PSA module facilitates the fusion of features from different scales and locations, enhancing the model’s capacity to understand and process intricate image structures. The combination of these two modules can significantly enhance the model’s focus on important regions, thereby improving the accuracy and robustness of the model’s segmentation performance.

**Fig 5:**
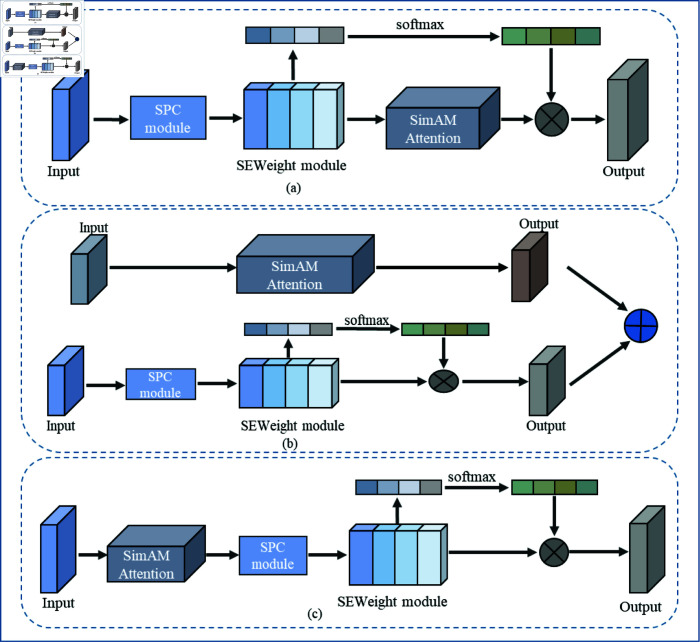
Attention module structure in three combinations (a) tandem, (b) parallel, (c) nested.

We combined the SimAM and PSA attention modules in series, parallel, and nested configurations, integrating them into UNet to create the TandemAT-UNet, ParallelAT-UNet, and NestedAT-Unet models for training. This approach aimed to validate their effectiveness and explore the optimal combination, with the structure of the combined attention modules illustrated in Fig 5. The serial structure enhances feature representation incrementally by successively applying the SimAM and PSA modules at different levels, thereby improving both local and global features of the image. This layer-by-layer feature enhancement mechanism aids in increasing the segmentation accuracy of the UNet network, particularly in capturing the details and edges of the skin images. The parallel structure allows the network to more comprehensively capture local and global information, further improving segmentation accuracy for the details and edges of skin images. Nesting the SimAM module within the PSA module creates a nested structure that effectively suppresses irrelevant features and noise during PSA multi-scale feature extraction, thereby enhancing the fineness of the features.

#### Evaluation metrics.

To compare the segmentation performance of the four models—ParallelAT-UNet, TandemAT-UNet, NestedAT-UNet, and UNet—four evaluation metrics were employed: Dice coefficient (1), accuracy (2), mean Pixel Accuracy (mPA) (3) and mean Intersection over Union (mIoU)) (4).

The Dice coefficient is a commonly used metric for measuring the similarity between two samples and is particularly suitable for medical image segmentation. Its value ranges from 0 to 1, where 1 indicates complete overlap and 0 indicates no overlap at all; a higher value signifies better segmentation performance. The formula for the Dice coefficient is as follows:

$$Dice=\frac{2|X\cap Y|}{\left|X|+|Y\right|}$$ (1)

where X and Y denote two sample sets, respectively, and |X∩Y| denotes their intersection. In image segmentation, X usually denotes the predicted segmentation region and Y denotes the true segmentation region. Accuracy is the ratio of the number of samples correctly classified by the model to the total number of samples, and is often used together with other evaluation metrics to comprehensively evaluate the model performance.

$$accuracy=\frac{TP+TN}{TP+FP+FN+TN}$$ (2)

Where TP is the true class, TN is the true negative class, FP is the false positive class, FN is the false negative class. MPA is used to measure the pixel categorization accuracy of each class and then the average of these accuracies is calculated. MPA can be denoted as:

$$mPA=\frac{1}{k+1}\sum_{i=0}^{k}\frac{p_{ii}}{\sum_{j=0}^{k}p_{ij}}$$ (3)

It can measure the impact of each category. MIoU is a standard measure of semantic segmentation, IoU is usually used to measure the degree of overlap between predicted and real regions. MIoU is averaged over all categories of IoU and can be expressed as:

$$mIoU=\frac{1}{k+1}\cdot \sum_{i=0}^{k}\left(\frac{p_{\left(i,i\right)}}{\sum_{j=0}^{k}p_{\left(i,j\right)}+\sum_{j=0}^{k}p_{\left(j,i\right)}-p_{\left(i,i\right)}}\right)$$ (4)

Where i denotes the true value and j denotes the predicted value. Then p(i,i) denotes true positive, p(i,j) denotes true negative, p(j,i) denotes false positive, and p(j,j) denotes false negative.

Floating-point operations (FLOPs) refers to the number of floating-point operations required to run a network model once, and is a measure of how many floating-point operations need to be performed by the model during a single forward propagation. FLOPs are often used as a measure of the computational efficiency and speed of a model.

Params, the number of parameters to be learned in the model, is another important measure of model complexity. More GPU memory is required when training the model.

#### Training.

The experiments were performed in a hardware environment with device Intel Xeon W-2255 CPU and Nvidia RTX A5000 GPU, and the software environment was based on Python 3.9 and PyTorch 1.11.0 framework (CUDA11.3,cuDNN8.2.0). Training was performed using a weighted cross-entropy loss function, with weights of 1.5 and 1.0 assigned to the damage region and the background, respectively, to address the category imbalance. The optimizer was chosen to be Adam, with an initial learning rate of 1e-4 and L2 regularization was imposed (weight decay factor of 1e-4) with a period of 50 epochs. the training process lasted for 200 epochs, with a batch size of 4. Due to the limitation of the video memory capacity, gradient cropping (maximum paradigm 1.0) was also captured to prevent gradient explosion. The dataset is divided into training, validation and test sets according to 8:1:1.

### Damage calculation

To assess the changes in mouse skin damage after different doses of laser irradiation, this study employed a quantitative analysis method based on 3D reconstruction. The damage volume of skin tissue was calculated by analyzing the B-Scan images obtained from OCT scanning pixel by pixel. In the experiment, a 1 cm × 1 cm × 0.5 cm 3D area of skin was scanned using OCT, generating 200 B-Scan images with a resolution of 460 × 500 pixels, with each image representing a cross-section of skin. The boundary contours of the damaged areas were extracted by performing 3D reconstruction on the segmented images using the VTK module. The damaged volume of the skin region was determined by counting the number of pixels representing the damaged area in each B-Scan image and accumulating the damaged pixels across all slices. This accumulated pixel count was then multiplied by the volume of each pixel. The calculation formula is as follows Eq (5):

$$V=V_{P}\times \sum_{k=1}^{m}F(s_{k})$$ (5)

where V represents the total volume of the damaged region and VP represents the volume of each pixel. In this experiment, each voxel has a size of 10 μm × 22 μm × 50 μm and a volume of $11,000\;\mu {\text{m}}^{3}$. F(s_*k*_) is the function used to identify the damaged pixels in each B-Scan image slice. This quantitative assessment method for laser-induced skin damage was utilized to elucidate the effects of different doses on skin damage and its healing over time.

## Results

### Analysis of skin OCT image data enhancement effect

**Fig 6:**
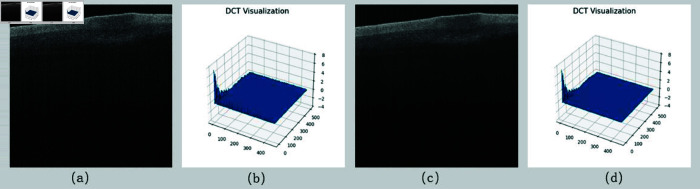
(a) The original image of SS-OCT scanned skin; (b) The corresponding frequency domain value map of the original image; (c) The corresponding noise reduction result map of the original image; (d) The frequency domain value map of the noise reduction map.

OCT images are typically affected by scattering noise, leading to a blurred image hierarchy and details, which impairs the recognition of skin tissues. As shown in Fig 6a, the unprocessed image exhibits low contrast, making it difficult to define the boundaries of the skin layers, with obvious noise interference. To improve image quality, we preprocessed the original image using BM3D denoising. As illustrated in Fig 6c, the overall clarity of the image was significantly enhanced, with the boundary lines of the skin structures becoming more distinct and the contours of the damaged areas clearer. The noise reduction effect was validated using frequency domain maps, as depicted in Figs 6b and 6). The frequency domain maps after denoising displayed fewer noise components and more concentrated spectral energy compared to those before denoising, indicating that the denoising algorithm effectively suppressed high-frequency noise components in the image. Consequently, image preprocessing using the BM3D denoising method provides more reliable image data for subsequent deep learning network input.

### Deep learning qualitative segmentation results

**Fig 7:**
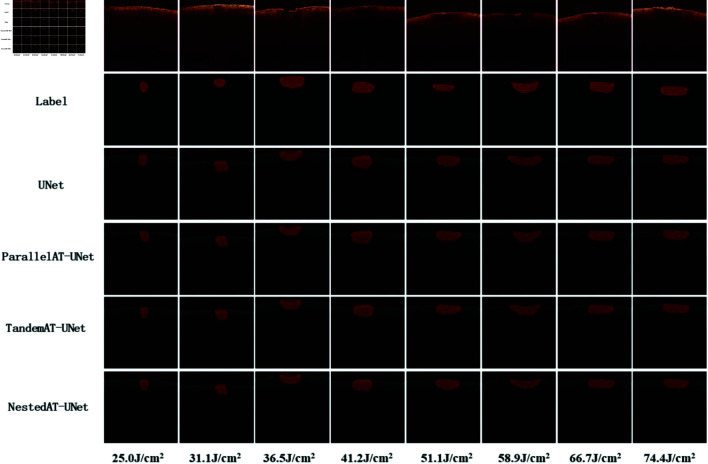
Segmentation results of SS-OCT scans after three days of irradiation at different doses.

The results of the automatic segmentation of skin OCT images in the test set using four network models—UNet, ParallelAT-UNet, TandemAT-UNet, and NestedAT-UNet—are presented in Fig 7. All three enhanced attention networks, along with UNet, successfully segmented the laser-induced skin damage regions. A comparison of the segmentation results from the four models revealed that ParallelAT-UNet achieved the most accurate segmentation and exhibited the highest overall segmentation performance. The results from the nested structure network were similar to those of the tandem combined attention network; however, the segmentation accuracy of the nested structure was slightly inferior to that of the tandem structure. Among the models, the unimproved UNet network displayed the poorest segmentation performance.

The first row shows eight representative B-scan images from the test set, the second row shows the manually labeled images, and the third to sixth rows show the segmentation results of U-Net, ParallelAT-UNet, TandemAT-UNet, and NestedAT-UNet, respectively.

### Quantitative analysis of network model segmentation performance

The performance of the four network models was comparatively analyzed using manually labeled images as references before and after noise reduction. The prediction results of the network models were evaluated on the test set, as shown in Table 1. The segmentation results of the image data after noise reduction in all four network models were significantly better than those of the images without noise reduction. Additionally, compared to the base UNet model, the segmentation performance of the three networks that incorporated serial, parallel, and nested combined attention modules improved for both pre- and post-noise-cancelled image data. Among these, the ParallelAT-UNet demonstrated the best segmentation performance for the post-noise-cancelled skin OCT image data, with evaluation metrics of Dice, mIoU, mPA, and accuracy reaching 0.9364, 92.67, 96.31, and 99.39, respectively.

**Table 1:** Evaluation metrics results (Dice, mIoU, mPA, and accuracy) of the four network models on the test set before and after denoising (bold indicates best results).

Method	Pre-noise				Post-noise			
	Dice	mIoU (%)	mPA (%)	Accuracy (%)	Dice	mIoU (%)	mPA (%)	Accuracy (%)
TandemAT-unet	0.9168	90.05	95.43	99.05	0.9261	92.62	95.99	99.36
ParallelAT-UNet	0.9224	89.54	96.12	99.12	0.9364	92.67	96.31	99.39
NestedAT-UNet	0.9264	89.63	95.31	99.08	0.9206	92.62	95.46	99.32
UNet	0.8926	88.79	92.18	99.06	0.9137	92.17	95.50	99.34

To further validate the performance effect of ParallelAT-UNet, we compare it with the classical attention modules CBAM and SE. The results, as shown in Table 2, indicate that ParallelAT-UNet outperforms the conventional method in all four metrics. This suggests that the parallel combination of SimAM and PSA can more efficiently fuse local details with global contextual information, thus improving the boundary recognition of complex damage.

**Table 2:** Additional evaluation metrics results (Dice, mIoU, mPA, and accuracy) including UNet-CBAM and UNet-SE.

Method	Dice	mIoU (%)	mPA (%)	Accuracy (%)
UNet-CBAM	0.8937	91.56	93.81	98.93
UNet-SE	0.9049	90.52	94.44	99.27
ParallelAT-UNet	0.9364	92.67	96.31	99.39

For a fair comparison, we migrate three SOTA methods, SWIN-UNet , PVT-CASCADE , and TransCASCADE, to our dataset and evaluate their performance in the same training and testing environment. Meanwhile, we maintain consistent data preprocessing methods and evaluation metrics, including Dice, mIoU, mPA, and Accuracy. In addition, we supplement the model efficiency-related metrics (FLOPs, number of parameters) to fully demonstrate the performance differences. Results are shown in Table 3. The ParallelAT-UNet model demonstrates exceptional performance across four metrics: Dice, mean Intersection over Union (mIoU), mean Pixel Accuracy (mPA), and Overall Accuracy. Additionally, it has 44.45 FLOPs and 30.66 parameters, significantly surpassing the results of the three other state-of-the-art (SOTA) methods on this dataset.

**Table 3:** Evaluation metrics results (Dice, mIoU, mPA, accuracy, FLOPs, and Params) of various models.

Method	Dice	mIoU (%)	mPA (%)	Accuracy (%)	FLOPs (G)	Params (M)
ParallelAT-UNet	0.9364	92.67	96.31	99.39	44.45	30.66
SWIN-UNet	0.8992	89.95	92.76	99.02	51.36	34.72
PVT-CASCADE	0.9153	90.99	94.83	99.15	46.23	34.93
TransCASCADE	0.9285	92.46	95.37	99.28	46.72	32.19

### Assessment of damage healing in mice

**Fig 8:**
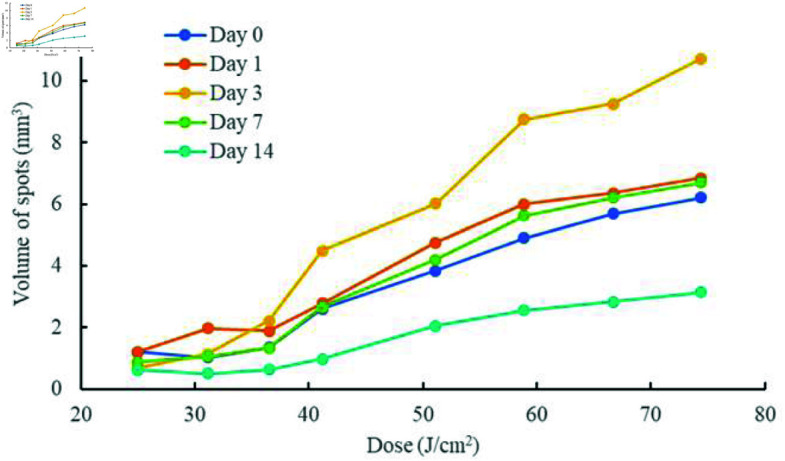
Damage healing results with time and dose.

Damage segmentation was performed on OCT images of mouse skin laser damage using the ParallelAT-UNet model, followed by the calculation of damage volume through a 3D reconstruction method. The effect of different laser doses on mouse skin damage volume was quantitatively assessed, as shown in Fig 8.

On the day of laser irradiation and from 1 to 14 days post-irradiation, the damage volume of mouse skin increased significantly with the increasing laser irradiation dose. Within the range of doses from $31.1\;{\text{J/cm}}^{2}$ to $74.4\;{\text{J/cm}}^{2}$, the damage volume exhibited a trend of initially increasing and then decreasing, reaching a maximum value 3 days after irradiation. By 7 days post-irradiation, the damage volume was close to the initial irradiation volume, and by 14 days post-irradiation, the damage volume was significantly lower than the initial damage volume. These findings indicate that while the laser skin tissue damage in mice showed notable healing by 14 days post-irradiation, it did not completely recover to normal tissue.

## Discussion

The application of neural networks in medical skin image segmentation has become increasingly widespread and intensive in recent years. While the traditional UNet network performs well in segmenting and evaluating normal skin medical images, research on segmenting and evaluating damaged skin images remains deficient. In this study, we acquired OCT image datasets of skin injuries in mice subjected to different doses of 1 μm -2 μm lasers using an SS-OCT imaging system. To address these deficiencies, we first applied data enhancement techniques by introducing the BM3D denoising algorithm to preprocess the OCT images. The results demonstrated that this approach effectively improved image quality and visual clarity. The denoising process not only significantly enhanced image clarity and made the contours of the damaged regions more distinct but also laid the groundwork for subsequent network segmentation and quantitative analysis of the dataset formed through denoising preprocessing.

Moreover, the widespread application of the attention mechanism in deep learning has shown significant advantages. By focusing on key regions in the image, the attention mechanism can effectively improve segmentation accuracy. To further address these shortcomings, this study designed a convolutional neural network that combines attention modules by integrating SimAM and PSA attention modules into the UNet upsampling process through various combinations of series, parallel, and nested structures, resulting in three network configurations: TandemAT-UNet, ParallelAT-UNet, and NestedAT-UNet. Utilizing UNet and the three combinations of network structures for the segmentation of the laser skin damage OCT image dataset, the research results indicated that ParallelAT-UNet achieved the best segmentation performance, with a Dice coefficient, mIoU, mPA, and accuracy of 0.9364, 92.67%, 96.31%, and 99.39%, respectively, thereby validating the effectiveness of the attention mechanism. In summary, ParallelAT-UNet successfully realized the segmentation of laser skin damage OCT images, providing an effective means for subsequent quantitative research on laser skin damage.

Despite the results achieved in this study, some limitations still exist. First, the dataset in this study was small and limited to the range of specific laser doses and recovery times, which may limit the model’s generalization ability.and will be applied in the future to validate the model’s generalizability to other publicly available OCT datasets of damaged skin or images of skin damage caused by other laser irradiation doses. Future applications will be made to other publicly available damaged skin OCT datasets or other images of skin damage caused by laser irradiation doses to validate model generalizability.

The ParallelAT-UNet model was utilized to segment the pre-processed dataset of mouse skin damage OCT images taken from 0 to 14 days post-irradiation at doses ranging from $25.0\;{\text{J/cm}}^{2}$ to $74.4\;{\text{J/cm}}^{2}$. Subsequently, a three-dimensional reconstruction method was employed to quantitatively assess the volume of skin damage at different doses and recovery times, further analyzing the relationship between changes in skin damage volume and laser dose as well as recovery time. The results indicated that both laser dose and recovery time significantly influenced the extent of injury. Specifically, laser irradiation at doses of $25.0\;{\text{J/cm}}^{2}$ to $36.5\;{\text{J/cm}}^{2}$ did not result in significant changes in the volume of skin injury with respect to dose and recovery time. This may be attributed to the less severe nature of low-dose laser damage and the faster healing rates observed in the mice. In contrast, within the dose range of $44.2\;{\text{J/cm}}^{2}$ to $74.4\;{\text{J/cm}}^{2}$, the changes in damage degree relative to dose and recovery time were pronounced compared to the lower doses; the volume of skin damage increased significantly from 3 to 7 days after irradiation compared to the volume on the day of irradiation, indicating that higher laser doses had a secondary damaging effect on the skin. By 14 days post-irradiation, the volume of skin damage in each dose group had significantly decreased, with the mice exhibiting good healing of the skin damage and demonstrating a clear dose-effect relationship. In summary, the ParallelAT-UNet model, combined with three-dimensional reconstruction, achieved accurate segmentation and quantitative assessment of the damaged areas in mice, providing a viable approach for the quantitative evaluation of the degree of laser skin damage.

In summary, this study established a 1 μm–2 μm laser mouse skin damage model and acquired skin damage OCT image data using an SS-OCT imaging system. The high-resolution dataset was preprocessed using the BM3D denoising algorithm, and the effects of TandemAT-UNet, ParallelAT-UNet, NestedAT-UNet, and UNet on the segmentation of the skin damage area were explored. Among these, ParallelAT-UNet emerged as the best performer, achieving an accuracy of 99.39%. Furthermore, the volume of skin damage was quantitatively evaluated using ParallelAT-UNet in combination with the three-dimensional reconstruction method, clarifying the relationship between dosage effects and recovery time. These results provide an effective means for the non-invasive evaluation of laser skin damage via OCT in clinical practice and offer an experimental basis for expanding the clinical application of lasers. It is important to note that individual differences in laser injury response and tissue repair abilities may impact the accuracy of the assessment. Future studies should incorporate physiological parameters or biological tissue markers for a multilevel functional assessment to gain a deeper understanding of the biological effects of laser injury. Additionally, while high-resolution OCT imaging was utilized, future research should consider integrating other imaging techniques or multimodal approaches to further enhance the sensitivity and specificity of injury monitoring. These considerations provide new directions for subsequent studies and contribute to achieving more accurate damage assessments and treatment effect evaluations in the field of laser medicine.

## Conclusion

In this study, we propose a quantitative analysis method using the UNet-AT model to evaluate changes in mouse laser skin injury based on different dosages and recovery time conditions. The experimental results show that the BM3D denoising algorithm can effectively suppress the high-frequency noise components in the SS-OCT images of skin injury, thereby significantly improving the clarity of the pictures and the performance of subsequent segmentation tasks. We designed three improved models, TandemAT-UNet, ParallelAT-UNet, and NestedAT-UNet, by integrating the SimAM and PSA attention modules into the up-sampling process of UNet in series, parallel and nested forms, respectively. All these models show high accuracy in the segmentation task of skin damage images, among which ParallelAT-UNet achieves the best segmentation performance with a Dice coefficient of 0.9364, mIoU of 96.31%, mPA of 92.67%, and accuracy of 99.39%. In the comparison experiments with the recent SOTA method, the ParallelAT-UNet model with FLOPs and Params of 44.45 G and 30.66 M performed optimally in the laser-induced mouse skin damage image dataset.

Further quantitative analysis showed that laser dose and recovery time significantly affected skin injury volume. No significant change in skin injury volume was observed at laser irradiation doses ranging from $25.0\;{\text{J/cm}}^{2}$ to $36.5\;{\text{J/cm}}^{2}$, whereas at higher doses ranging from $44.2\;{\text{J/cm}}^{2}$ to $74.4\;{\text{J/cm}}^{2}$, the injury volume changed significantly with increasing dose and recovery time. In addition, all dose groups achieved different degrees of healing 14 days after laser treatment, and the damage volume was significantly reduced from the initial volume. The results of this study not only verified the close relationship between laser dose and changes in skin damage and provided a reference for the individualized optimization of laser treatment parameters. In future clinical integration, individual differences in patient skin tissue and treatment safety should be fully considered to ensure the broad applicability of this method.

In addition, although this study utilized unimodal SS-OCT images for analysis, in clinical practice, multimodal imaging (e.g., ultrasound, biomicroscopy) may provide more comprehensive diagnostic information. In the future, we plan to extend the applicability of our method, including the introduction of more types of skin injury data and multimodal imaging analyses, to improve the generalizability and adaptability of the model. To verify the actual deployment potential of the model, we tested the inference speed of ParallelAT-UNet on an NVIDIA Jetson Nano. The inference time of the original model for a single image is 0.23 s. After channel pruning and compression, the amount of parameters is reduced by 30%, the inference time is lowered to 0.2 s, and the Dice coefficient is only reduced to 0.9247. This shows that the model can be adapted to edge computing devices through lightweighting techniques to meet clinical real-time requirements. In addition, we plan to develop a lightweight mobile-based version for real-time injury monitoring via TensorRT acceleration. Meanwhile, we will further optimize the model structure and algorithm efficiency to achieve higher segmentation accuracy and lower computational complexity, as well as better real-time performance to provide stronger support for clinical diagnosis and treatment and applied research.

In summary, the method proposed in this study combines efficient denoising processing, the introduction of the attention mechanism, and quantitative analysis of damage to provide an accurate and reliable tool for quantitatively evaluating the extent of laser skin damage and its changes with time and dose. The results of this study not only contribute to an in-depth understanding of the mechanism of laser action on skin damage but also provide an important scientific basis for the optimization of laser therapy.
